# Supplementary material for: Non-microbial methane emissions from tropical rainforest soils under different conditions
Source: PLoS One. 2021 Aug 5;16(8):e0255725. doi: 10.1371/journal.pone.0255725 (PMC8341527; doi:10.1371/journal.pone.0255725)
Supplement: Attachment — Submitted filename: Response to Reviewers.docx [file pone.0255725.s001.docx]

RE: **PLOS ONE**

Manuscript Number: PONE-D-20-33065

Title: “Non-microbial methane emissions from tropical rainforest soils under different conditions”

Dear Editor and Reviewers:

We would like to express our sincere gratitude to you and members of the editorial board for giving us the opportunity to revise our manuscript entitled “Non-microbial methane emissions from tropical rainforest soils under different conditions” (PONE-D-20-33065). We are also grateful for yours and the reviewers’ comments and suggestions which have helped us to improve the manuscript.

Appended to this letter is our point-to-point responses to the comments raised by your esteemed reviewers. We agreed with all comments raised by reviewers. We would like to take this opportunity to express our sincere thanks to the reviewers who identified some points in our manuscript that needed modification and correction. Based on the comments raised by reviewers, we were asked to explain and clarify some parts of our work which are blue below. Accordingly, we have uploaded a copy of the revised manuscript including all the changes made during the revision process highlighted with blue color for rapid identification. We hope that the revised manuscript is accepted for publication in Journal.

We are really looking forward to hearing from you soon at your convenience. Thanks for your time and consideration again.

Best regards,

Sincerely yours,

Wenjie Liu

30/06/ 2021

Reviewer #1: This incubation experimental study shows variations of NM-CH_4_ emission from tropical rainforest soils under different environmental conditions. The results confirm, as conclusively stated by the authors, the production mechanism of NM-CH_4_ emission by thermal degradation and reactive oxygen species oxidation. However, I’m expecting new findings beyond the results in previous studies except for the blank filling with new soil samples of rainforests in this study. Some other concerns are of the experimental treatments in the incubation.

1. Before other experiments, the soil NM-CH_4_ emission characteristics in the first 192 h were studied from incubation experiments in aerobic and anaerobic environments at 70 °C with natural soil water content (Line 127-129). The temperatures up to 70 °C are not naturally realistic in forest soils, please explain your rationale of making the experimental temperatures (Line 133) beyond natural realities.

**Author’s Answer:** Our research and previous series of studies (Wang et.al.2013; Gu et al.2015) have shown that high temperature can effectively stimulate the emission of NM-CH_4_ from soil. Under natural conditions, the annual average temperature of tropical primary forests is relatively low (in this study area the annual average temperature is 24.5℃ in this study area), so the emission of NM-CH_4_ from the soil is very weak in this condition. In order to explore the effect of moisture on NM-CH_4_ emission from the mechanism, we need to stimulate the soil NM-CH_4_ emission as much as possible when conducting indoor cultivation experiments. Therefore, this study chose to conduct the experiment at 70°C to deeply discuss the influence of water on soil NM-CH_4_ emission.

2. The anaerobic environment was created by blowing high-purity nitrogen in the experiment, the same as in Wang et al. (2011b) (Line134-135). In the study of Wang et al (2011b), they treated plant tissues and the anaerobic condition can be reasonably created and maintained by blowing high purity nitrogen. But in soil samples, there are tiny pores that trap oxygen in soil aggregations. I wonder the efficiency of erasing the oxygen in soil samples by merely blowing high-purity nitrogen. Some supplementary treatments are needed to ensure or check the anaerobic condition after blowing nitrogen.

**Author’s Answer:** The formation of an anerobic soil environment by blowing in high-purity nitrogen is based on the method used by Wang et al. (2011b). Although this method was first applied in the experiment of processing plant tissues, Wang et al. (2013) and Gu et al. (2015) also referred to this method to form an anaerobic environment when conducting soil NM-CH_4_ emission studies, indicating that this method is also applicable to soil cultivation experiments.

3. What’s the situation of 200% soil water content (Line 138)?

**Author’s Answer:** In the experiment, the water content of different gradients is mainly used to simulate the water environment under natural conditions. The reason why the experiment was conducted under the condition of 200% water content is that tropical forests tend to accumulate water in low-lying areas after heavy rains, resulting in differences in the degree of soil flooding.

4. The experimental results showed that the soil would no longer release NM-CH_4_ when the incubation time was 229 h (Line 180). The degradation of soil organic matters, which is the source of NM-CH_4_ production and emission, is a long term process up to hundreds to thousands years. Without mechanical explanation of the experimental phenomena, it’s hard to believe that the long term continuous degradation of SOM produces no NM-CH_4_ after 229h.

**Author’s Answer:** Indeed, as you said, the degradation of soil organic matter is a continuous process, which provides a continuous substrate for the emission of soil NM-CH_4_ due to the presence of microorganisms, so soil NM-CH_4_ will not end in a short period of time. However, the indoor simulation experiment in this study does not involve the participation of microorganisms. This cuts off the process of continuous degradation of organic matter by microorganisms, so there is no substrate supplement for NM-CH_4_ emission. Therefore, after 229 hours, the substrate for NM-CH_4_ emission is exhausted, and we can believe that the soil will no longer release NM-CH_4_ at this time.

5. Fig. 5 shows that the NM-CH_4_ emission increased when soil water contents rose from 0% to 5%, and thereafter, more soil water content inhibited significantly the NM-CH_4_ emission. It seems that water molecules play key roles in the process of NM-CH_4_ production in soils. Could the authors explain it in the context of organic chemistry?

**Author’s Answer:** Thanks, the question you raised is very professional and important. After in-depth thinking and reviewing of the existed literature, we found that it is difficult to explain this phenomenon from the perspective of organic chemistry based on the current and our research results. It may be caused by the role of hydrogen bonds in water. However, as the soil water contents increased from 5% to 10%, this roles decreased.

6. The total soil NM-CH_4_ emission fluxes in the tropical rainforest were higher than those in the tropical rubber forest (Line 232-234). This implies that the types of ecosystems (may also be the chemical composition of SOM in ecosystems) are key factors in NM-CH_4_ emission. I recommend some discussion of the results in this study to the published literatures (e.g. Wang et al., 2013).

**Author’s Answer:** Thank you for your suggestion. The article has been revised and the related research results have been added in discussion. Corresponding change is located in line 291-297 of the revised manuscript.

“When conducting experiments on NM-CH_4_ emission from aggregates, Wang et al. found that there is a significant positive correlation between the NM-CH_4_ emission rate of soil aggregates and the concentration of organic carbon, indicating that the role of the amount of organic carbon can determine soil NM-CH_4_ emissions [10]. Similarly, the studies of Gu et al. and Hurkuck et al. also reported that there is a significant positive correlation between the release rate of soil NM-CH_4_ and the soil organic carbon content [23,24]. It supported that soil NM-CH_4_ was derived from soil organic matter. But what needs to be further clarified is which organic molecules or structures in the soil are involved in the process of soil NM-CH_4_ release. In the study of plant materials, it is found that the methoxy groups in pectin and lignin can be used as precursors of NM-CH_4_ production [18,20], which provides ideas for the study of soil NM-CH_4_ sources.”

Reviewer #2: Manuscript ID: PONE-D-20-33065
Title: Nonmicrobial methane emissions from tropical rainforest soils
Recommendation: Major revision
Comments:
1. Title: The title seems not complete, need to be more specific to your research.

**Author’s Answer:** Thank you for your suggestion. The title has been revised to "Non-microbial methane emissions from tropical rainforest soils under different conditions"

2. Keywords: add one keyword: “tropical rainforest soils”

**Author’s Answer:** The keyword "Tropical Rainforest Soil" has been added of the manuscript.

3. Abstract

• State the aim and the novelty of the study.

**Author’s Answer:** The research purpose and novelty have been stated in the abstract line 16-17 of the revised manuscript. “In order to explore the laws and characteristics of NM-CH_4_ emission from tropical rainforest soils.”

4. Materials and methods

• Citations needed. Please cite the papers of where the methods are adapted from.

**Author’s Answer:** Citations have been added, please see the article line 157 for details

“26.	Yang H, Liu S, Li Y, Han X. Diurnal variations and gap effects of soil CO_2_, N_2_O and CH_4_ fluxes in a typical tropical montane rainforest in Hainan Island, China. Ecol Res. 2017; 33: 379-392. https://doi.org/10.1007/s11284-017-1550-4.”

5. Incubation experiment:
• Why choose the first 192h for incubation experiments?

**Author’s Answer:** We have done some pre-experiments, and we have found that the NM-CH_4_ emission rate presented a logarithmic function as the incubation time increase. From the function, the NM-CH_4_ emissions rates decreased as a constant in 192 h. Finally, we choose the first 192 h for incubation experiments.

6. • Why choose these 8 gradients, 0%, 5%, 10%, 30%, 50%, 70%, 100%, and 200%? Same question for “The mass concentrations of H2O2 were set at 0%, 0.1%, 0.25%, 0.5%, 1%, and 2% “

**Author’s Answer:** The moisture and hydrogen peroxide gradient culture experiment mainly referred to the experimental treatment of Wang et al. (2013). The main purpose is to explore whether the influence law of water and hydrogen peroxide on NM-CH_4_ emission is also applicable in tropical forest soils.

7. • Line 146, (table 1), should be Table 1.

**Author’s Answer:** Thank you for your correction. We have done this work.

8. CH4 concentration measurements:

• Does this method part refer to any reference?

**Author’s Answer:** Thank you for your suggestion. The related literature has been cited. Please see line for 157 details.

9. Results and discussions

• Line 285-286: “More research is needed on the mechanisms of soil water content affecting NM-CH_4_ emissions in the future.” Please clarify which specific research can be done, give your suggestions.

**Author’s Answer:** Thank you for your suggestion. Related content has been added to the article. Please see line 285-287 for details.

“For example, what is the internal mechanism of moisture affecting soil NM-CH_4_ emission, how to explain its internal mechanism at the level of organic chemistry, and where is the critical point of the impact of moisture on soil NM-CH_4_ emission?”

10. • Line 298-301: “How to conduct field in situ observations, intuitively estimate its proportion to total CH4 emissions, and explore more emission substrates and emission paths still needs to be further studied.”Can you give some solutions in response to this problem? E.g. What methods can be conducted to solve this problem?

**Author’s Answer:** Thank you for your suggestion. Related content has been added to the article. Please see line 323-334 for details

“However, the current researches on soil NM-CH_4_ mainly concentrated on indoor incubation. How to conduct field in-situ observation (For example, two gaseous chemical reagents, monochloromethane and difluoromethane, are often used as inhibitors of CH_4_ producing bacteria and CH_4_ oxidizing bacteria, respectively. Can the two gases can be directly used in the field and to achieve the purpose of inhibiting biomethane, thereby determining the release rate of NM-CH_4_ under natural conditions), intuitively estimate its proportion to total CH_4_, and explore more emission substrates and emission paths (For example, can the emission substrate and path be determined by the method of element labeling) still need to be further studied. This study found that temperature and moisture have a significant impact on NM-CH_4_ emissions. The high-temperature and high-humidity soil environment of tropical forests, coupled with abundant organic carbon storage, can be considered as the best site for in-situ observation of microbial CH_4_ and NM-CH_4_.”

11. Conclusion

• Please state the importance of the study and how it can contribute to the research community.

**Author’s Answer:** Thank you for your suggestion. Related content has been added to the article. Please see line 321-323 for details. “In-depth research on NM-CH_4_ plays an important role in further understanding the emission mechanism of soil CH_4_ and more accurate prediction of climate change.”

12. • Please include the detailed limitations and what can be done for the future studies

Conclusion

**Author’s Answer:** Thank you for your suggestion. Related content has been added to the article. Please see line 335-341 for details.

“As we all know, model simulation is the best way to predict the current global change trend. If the process of NM-CH_4_ is ignored, it may further increase the uncertainty of the regional and global carbon cycles predicted by current biogeochemical models. Therefore, based on the research that can distinguish between microbial CH_4_ and NM-CH_4_, algorithms for the production and emission of NM-CH_4_ can be developed, verified, and integrated into the land surface model to better understand and predict large temporal and spatial changes.”
